# Controlling the fold: proprioceptive feedback in a soft origami robot

**DOI:** 10.3389/frobt.2024.1396082

**Published:** 2024-05-21

**Authors:** Nathaniel Hanson, Immanuel Ampomah Mensah, Sonia F. Roberts, Jessica Healey, Celina Wu, Kristen L. Dorsey

**Affiliations:** ^1^ Institute for Experiential Robotics, Northeastern University, Boston, MA, United States; ^2^ Department of Mathematics and Computer Science, Wesleyan University, Middletown, CT, United States

**Keywords:** origami, soft robotics, flexible electronics, feedback control, capacitive sensor, soft sensor, proprioception

## Abstract

We demonstrate proprioceptive feedback control of a one degree of freedom soft, pneumatically actuated origami robot and an assembly of two robots into a two degree of freedom system. The base unit of the robot is a 41 mm long, 3-D printed Kresling-inspired structure with six sets of sidewall folds and one degree of freedom. Pneumatic actuation, provided by negative fluidic pressure, causes the robot to contract. Capacitive sensors patterned onto the robot provide position estimation and serve as input to a feedback controller. Using a finite element approach, the electrode shapes are optimized for sensitivity at larger (more obtuse) fold angles to improve control across the actuation range. We demonstrate stable position control through discrete-time proportional-integral-derivative (PID) control on a single unit Kresling robot via a series of static set points to 17 mm, dynamic set point stepping, and sinusoidal signal following, with error under 3 mm up to 10 mm contraction. We also demonstrate a two-unit Kresling robot with two degree of freedom extension and rotation control, which has error of 1.7 mm and 6.1°. This work contributes optimized capacitive electrode design and the demonstration of closed-loop feedback position control without visual tracking as an input. This approach to capacitance sensing and modeling constitutes a major step towards proprioceptive state estimation and feedback control in soft origami robotics.

## 1 Introduction

Soft robots may soon be part of daily life, with envisioned applications ranging from helping humans (Robertson et al., 2021; Wu et al., 2021) to holding fragile objects without causing damage (Li et al., 2019). These robots currently have limited impact outside of the lab (Hawkes et al., 2021), but a growing body of work is demonstrating the state estimation, position control, and force control (Best et al., 2016; Tapia et al., 2020) that is essential for integrating soft robots into these target applications.

A wide range of approaches are used for position or force sensing; common types are motion capture (Della Santina et al., 2018; Patterson et al., 2020), pressure sensors external to the actuator (Best et al., 2016), and embodied mechanical sensors (Yan et al., 2023). Many scenarios in which feedback controlled soft robots are expected to excel over rigid robots, such as collaboration with humans or use in highly portable applications, present privacy or logistical challenges for motion capture. Pressure sensing is only possible for controlling fluidic actuators, and its accuracy will vary with leaks and tubing size. Additionally, range sensing on deformable objects is subject to interference and misalignments between sensors as the actuators deform. Soft mechanical sensors can both be integrated directly into the robot body and provide accurate force and shape estimations (Kim et al., 2021; Dorsey et al., 2022; Sun et al., 2022b; Dong et al., 2022; Thuruthel et al., 2019; Huang et al., 2022; Wang et al., 2022). Improving mechanical sensor performance to achieve closed-loop control will be critical to meet future soft robotics sensing and control needs.

**FIGURE 1:**
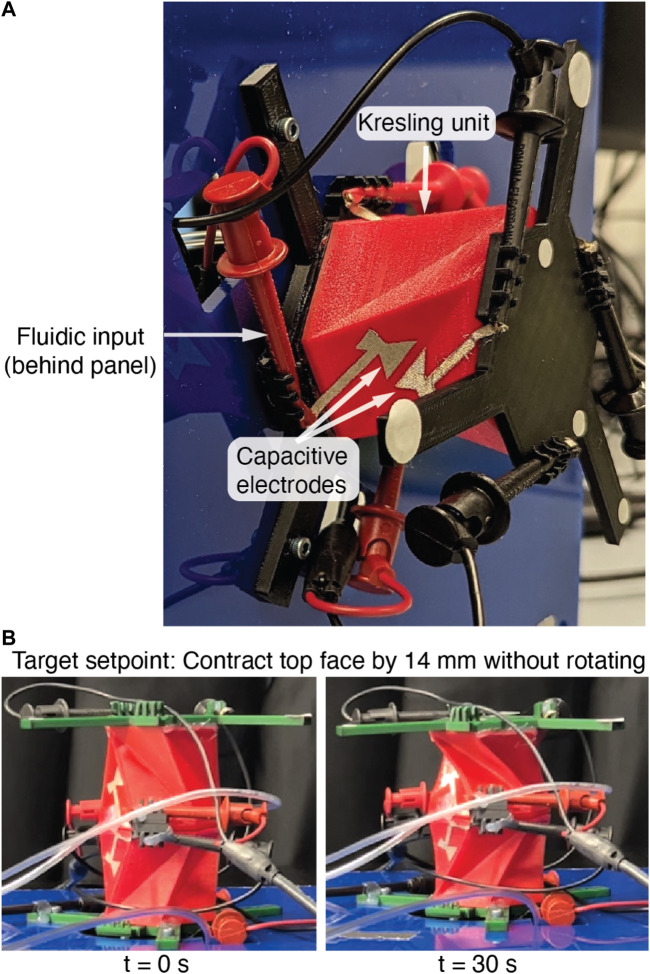
**(A)** The single-unit Kresling robot in the test stand. The capacitive sensors used for proprioception and feedback control are visible on one of three valley fold sets. The retroreflective markers are used only for optical groundtruthing. **(B)** The vertically stacked, two-unit Kresling robot during a static setpoint test, with target top face contraction of 14 mm with 0° rotation. In contrast to the single-unit robot, this robot has two degrees of freedom with independent contraction and rotation of the top face.

In this work, we demonstrate proprioceptive sensing and feedback control of the pose of single and two degree-of-freedom (DOF) Kresling (Kresling 2012) origami robots (Figure 1). Previous research in Kresling robots has assembled multiple units to form multi degree of freedom arms (Wu et al., 2021; Kaufmann et al., 2022) and crawlers (Pagano et al., 2017; Ze et al., 2022). These works showcased the potential of open loop controlled multi-unit robots to achieve extension, roll, and pitch at the face of each unit and translation and bending at the robot tip. However, closed loop operation will be necessary for operation in a range of environments with external forces and non-idealities.

This work investigates the design of capacitive sensors for robot proprioception and simple feedback control. We also show that the Kresling robot approach is compatible with the common soft robotic principles of elastomeric material fabrication and fluidic actuation. This work contributes to the state-of-the-art in soft robotics proprioceptive control by:

1. Designing capacitive position sensors to meet practical mechanical and electrical constraints for soft origami feedback control.
2. Fabricating 3D-printed elastomeric Kresling units to enable fluidic actuation.
3. Demonstrating feedback control of Kresling robots using only capacitive electrodes, including a two degree of freedom, two-unit robot with independent extension and yaw control.

Under fluid input of 80 kPA absolute, the single-unit actuator contraction is 75% of its total length (31 mm). We demonstrate single-unit feedback control to 40% of the extended length (17 mm) and error below 4 mm for all setpoints. In the two-unit robot, we demonstrate independent control of the contraction and rotation of the top face to 14 mm of contraction and ±16° of rotation. To our knowledge, this is the first demonstration of a soft origami robot with feedback control using only capacitive proprioception.

## 2 Related work

Shih et al. (2020) offer a comprehensive review of the state of soft robotic shape estimation, perception, and efforts towards closed loop control. Here, we highlight recent efforts in soft material sensors for actuator shape or force estimation using flexible circuit components such as capacitors (Kim et al., 2021; Dorsey et al., 2022), resistors (Sun et al., 2022b; Dong et al., 2022; Thuruthel et al., 2019), or inductors (Huang et al., 2022; Wang et al., 2022) fixed to the robot body. Detecting the angle between two actuator faces has also been well reported (Mori and Onoe, 2021; Wang et al., 2022), with angle accuracies ranging from 12° (Mori and Onoe, 2021) for a piezoresistive sensor to ±1° for an inductive sensor (Wang et al., 2022). Fold angle sensing is particularly advantageous in folded and origami robots, where the mechanical properties and deformation of an actuator may be designed through pattern selection and parameters (Park et al., 2022) and the relationship between fold angles and faces is well-defined.

Earlier demonstrations of origami robots folded in paper or polymers focused on the ability to reconfigure, deploy, or self-assemble by folding or unfolding (Robertson et al., 2021; Chen et al., 2022) using actuation approaches such as magnetic fields (Wu et al., 2021; Ze et al., 2022), fluidic pressure (Park et al., 2022; Chen et al., 2022), electric fields (Li et al., 2018), and tendons (Kaufmann et al., 2022). Insight gained from these fabrication, actuation, and sensing approaches spurred interest in more complex patterns and motions. Origami patterned robots have led to demonstrations as grippers (Robertson et al., 2021), crawlers (Chen et al., 2022; Sun et al., 2022a; Ze et al., 2022), jumpers (Sun et al., 2022b), and haptic feedback devices (Williams et al., 2022). One common design for origami soft robots is the Kresling pattern due to its ability to achieve bistable states defined by its pattern geometry and its behavior as a two DOF joint when compressed (Kresling, 2012; Kim et al., 2022; Babu et al., 2023). Prior work has applied the Kresling pattern to create crawlers (Ze et al., 2022; Pagano et al., 2017) and multi-segment continuum arms under magnetic (Wu et al., 2021) or cable-driven (Kaufmann et al., 2022) actuation.

In contrast to the wealth of soft mechanics and actuator work present in origami robotics literature, position sensing and feedback control research has received less attention. Notable recent work has investigated the integration of rigid (e.g., photoresistor) or soft mechanical sensors into active origami structures for on-off feedback control. Nisser et al. embedded phototransistors and LEDs in a flat origami structure to control angles during structure self-folding (Nisser et al., 2016). Researchers have also integrated contact switches and tactile sensors (Yan et al., 2023; Sun et al., 2022a) to enable hysteresis control in jumping, gripping, and crawling soft robots. The uses of soft sensors in this domain include measuring gait and environmental contact in an origami-patterned walker by coating the legs with piezoresistive material (Dong et al., 2022), estimating the curvature of a soft finger with a flexible inductor (Huang et al., 2022), and controlling the fold of a shape memory alloy in three states with a piezoresistive curvature sensor (Firouzeh and Paik, 2015).

## 3 Structure and modeling

### 3.1 Parameters and kinematic model

Previous work has thoroughly investigated the mechanics of the Kresling pattern (Kaufmann et al., 2022; Bhovad et al., 2019). Here, we present a short definition of properties relevant to the modeling of single Kresling unit and expand this model to a vertically stacked, two-unit Kresling structure with independent heights. Table 1 is a list of all variables and definitions.

**TABLE 1:** The Kresling unit properties.

Symbol	Meaning	Symbol	Meaning
*L*	Top length referenced to base	*α*	Top rotation referenced to base
*L*_*c*_	Full contraction length	*α*_*c*_	Full contraction rotation of top face
*L*_*e*_	Full extension length	*α*_*e*_	Full extension rotation of top face
*L*_*2DOF*_	2DOF top length referenced to base	*α*_*2DOF*_	2DOF top rotation referenced to base
*L*_*b*_	Center length referenced to base	*α*_*b*_	Center rotation referenced to base
*L*_*t*_	Top length referenced to center	*α*_*t*_	Top rotation referenced to center
*R*	Top face radius	*ϕ*	*π*/*N*
*N*	Number of top face edges	*ξ*	Sidewall valley fold angle
*λ*	Ratio of valley fold to base angle over *γ*	*γ*	*π*/2 − *π*/*N*

**FIGURE 2:**
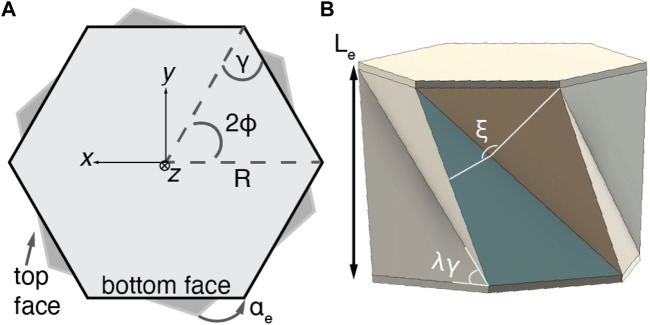
The parameters of the Kresling structure **(A)** from the bottom face looking up and **(B)** in 3D. The vector $\vec{n_{t}}$ is normal to the red shaded face, and the vector $\vec{n_{b}}$ is normal to the blue shaded face.

The single-unit structure has one DOF, as described by linked parameters *L* and *α*. Four parameters control the morphology: *L*_*c*_, *R*, *N*, and *λ*. Figure 2 represents these parameters on the Kresling structure with views (a) in 2D, looking up through the bottom face, and (b) in 3D. These variables are linked by Kaufmann et al. (2022).

$$L=\sqrt{L_{c}^{2}+2R^{2}\left[\cos\left(\alpha +2\phi \right)-\cos\left(\alpha_{c}+2\phi \right)\right]},$$ (1)

where *α*_*c*_ = 2*λ*(*π*/2 − *ϕ*).

We will refer to three additional dependent parameters to describe the Kresling structure. At full extension, the structure has length *L*_*e*_ and rotation *α*_*e*_. Angle *ξ* is linked to *L* by finding the angle of intersection between vectors normal to the sidewall triangular faces,

$$n_{t}=\left[\begin{array}{c} RL\sin\left(2\phi \right)\hat{i}\\ RL\left(1-\cos\left(2\phi \right)\right)\hat{j}\\ R^{2}\left(\sin\left(\alpha \right)\left(1-\cos\left(2\phi \right)\right)+\left(1-\cos\left(\alpha \right)\right)\left(\sin\left(2\phi \right)\right)\right)\hat{k} \end{array}\right]$$ (2)

and

$$n_{b}=\left[\begin{array}{c} RL\left(\sin\left(2\phi -\alpha \right)+\sin\left(\alpha \right)\right)\hat{i}\\ -RL\left(\cos\left(2\phi -\alpha \right)-\cos\left(\alpha \right)\right)\hat{j}\\ R^{2}\left(\left(\cos\left(2\phi -\alpha \right)-\cos\left(\alpha \right)\right)\left(\sin\left(2\phi -\alpha \right)+\sin\left(2\phi \right)\right)\right)\hat{k}-\\ R^{2}\left(\left(\cos\left(2\phi -\alpha \right)-\cos\left(2\phi \right)\right)\left(\sin\left(2\phi -\alpha \right)+\sin\left(\alpha \right)\right)\right)\hat{k} \end{array}\right]$$ (3)

where $\hat{i}$ , $\hat{j}$ , and $\hat{k}$ are unit vectors on the axes *x*, *y*, and *z*, respectively, such that

$$\cos\xi =\frac{{\vec{n}}_{t}\cdot {\vec{n}}_{b}}{\left\|n_{t}\|\|n_{b}\right\|}.$$ (4)

We derived the relationship between *L* and *ξ* through Eqs. 1−4. Observing that the relationship between fold angle and length is highly linear via a numerical approach (*R* ^2^ of 99.3% in the range of *L*_*c*_ to *L*_*e*_), we used a linear approximation for the inverse kinematics function

L=0.22ξ+10.4, (5)

where *ξ* is given in degrees.

The two-unit Kresling robot is composed of single units with independent fluidic inputs identical *L*_*c*_, *R*, *N*, and *λ* parameters, and variable lengths *L*_*t*_ and *L*_*b*_ for the top and bottom units, respectively. The two units are stacked vertically upon one another. The top unit is inverted from the bottom unit such that the center has rotation *α*_*b*_ = *α*_*e*_ and the top face has rotation *α*_*t*_ = −*α*_*e*_ when both top and bottom are fully extended. Actuating only the top unit contracts and rotates the top face clockwise, while actuating only the bottom unit contracts and rotates the top face counterclockwise. Actuating both units results in independent control of contraction and top face rotation with two degrees of freedom.

Taking the length from Eq. 1 for the top and bottom units, the length from base to top is

$$\begin{array}{ll} L_{\mathrm{2DOF}} & =\sqrt{L_{c}^{2}+2R^{2}\left[\cos\left(\alpha_{t}+2\phi \right)-\cos\left(\alpha_{c}+2\phi \right)\right]}\\ & \;+\sqrt{L_{c}^{2}+2R^{2}\left[\cos\left(\alpha_{b}+2\phi \right)-\cos\left(\alpha_{c}+2\phi \right)\right]}, \end{array}$$

while rotation of the top face is *α*_*2DOF*_ = *α*_*t*_ + *α*_*b*_.

### 3.2 Position sensors

Capacitive sensing offers straightforward signal readout with commercial measurement electronics and low hysteresis, but the placement of the electrodes must achieve high signal to noise ratio, high sensitivity near full extension, and robust operation under many extension-contraction cycles. A Kresling unit deforms by folding at the pattern hinges and contracting, so capacitive sensors could be located on the top and bottom faces (to measure gap) or the sidewall faces (to measure angle). Placing electrodes on the top and bottom faces would create a capacitor with a full extension gap of > 40 mm and result in a low signal to noise ratio near full extension. Electrodes placed on the sidewalls of the Kresling unit would increase signal to noise ratio over a top-bottom placement but require appropriate selection of shape, size, and orientation to fit on the triangular pattern faces and to maximize sensitivity.

Existing analytical approximations of the capacitance between angled plates (e.g., Seeger and Boser, 2003) typically assume the effect of fringing field is negligible and that a small angle approximation is valid. As the Kresling unit contracts and *ξ* decreases, the effect of the fringing field on total capacitance will shrink and the small angle approximation will not accurately describe the full range of contraction. Our initial tests also showed that the sidewalls flex and twist near the corners, which could cause delamination of the electrodes from the sidewall. Therefore, we simulated the capacitance change of several electrode shapes that did not cover the full sidewall using FEA and compared the sensitivity to a triangular electrode that covered the sidewall. We wished to model and compare the capacitance change that would occur by moving near full extension (i.e., *L*_*e*_) to the capacitance change that would occur by moving near full contraction (i.e., *L*_*c*_). Increasing the ratio of capacitance change near full extension over that of capacitance change near full contraction would improve the signal to noise ratio, so we optimized electrode shape to improve this ratio.

We optimized the electrode shape in COMSOL Multiphysics (Comsol Inc., v6.0) with a downhill simplex method (Lagarias et al., 1998) implemented in MATLAB (*fminsearch*, Mathworks, R2021a) through the Livelink for MATLAB toolbox (Comsol Inc., v6.0). This approach does not rely on an analytical solution of the gradient (e.g., Newton’s method) and was computationally feasible with FEA (e.g., vs. genetic algorithm).

**FIGURE 3:**
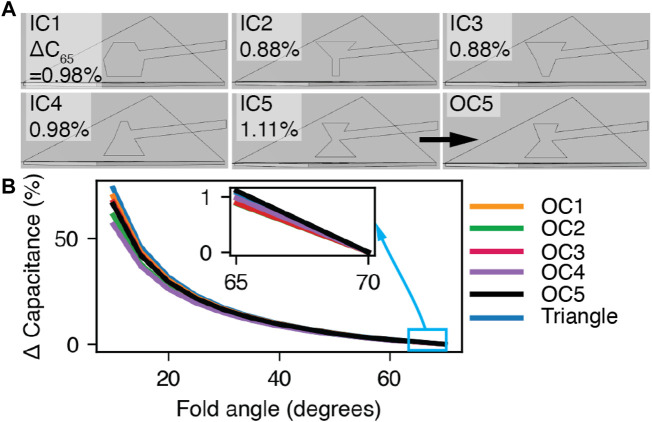
Finite element electrode modeling. **(A)** The initial electrode shapes IC1-IC5 and OC5. The optimized sensitivities are presented in the top left corners. **(B)** The simulation results for electrode shapes OC1−OC5 and the triangle electrode. *Inset:* Δ*C*_65_.

We constrained electrode size to the area of one triangular sidewall. Each electrode is constructed of the vertices in the subset *x*, which is a subset of all potential electrode points in $R^{2}$ bounded by the triangular sidewall face. Because the potential set of initial conditions is composed of all *n*-sided polygons, we constrained the number of vertices to five (to reduce model complexity) and the number of initial conditions to five. We chose five initial electrode shapes (ICs) of a hexagon (IC1), a funnel (IC2), triangular and inverted triangular shapes (IC 3–4), and an hourglass (IC5) (Figure 3A). These shapes spanned a large set of potential electrode shapes for optimization. To halve the model complexity, we enforced a symmetry condition on one sidewall, such that the angle between the triangular face and a horizontal plane is 0.5*ξ*. The 0.5*ξ* value at full extension is approximately 70°, and the value near full contraction is approximately 10°. We examined the ratio of capacitance change that would occur by moving 5° from full extension (i.e., Δ*C*_65_(*x*)) to the capacitance change that would occur at full contraction (i.e., *C*_10_(*x*)). The optimization function moved the location of the electrode vertices to maximize this ratio $f\left(x\right)=\frac{\Delta C_{65}\left(x\right)}{C_{10}\left(x\right)}$ .

Each initial condition reached an optimized condition (OC), and we selected OC5, the electrode with the largest $\frac{\Delta C_{65}\left(x\right)}{C_{10}\left(x\right)}$ , for experiments. Each $\frac{\Delta C_{65}\left(x\right)}{C_{10}\left(x\right)}$ is presented in Figure 3A. Figure 3B is a plot of the capacitance change $\frac{C-C_{70}}{C_{10}}$ across the full extension to contraction range for each OC and a triangular electrode that covers the sidewall (“triangle”). The Δ*C*_65_/*C*_10_ for the triangle electrode is 1.07%.

## 4 Materials and methods

### 4.1 Fabrication

To create an airtight chamber for fluidic actuation, we 3D printed the robot structure in thermal polyurethane (TPU). The TPU permits flexibility of the body and the requisite compliance to twist and contract the structure under the negative fluidic pressures that are commonly demonstrated in soft robotics. Moreover, the TPU is sufficiently nonporous, so it maintains a negative vacuum pressure well. The use of TPU limits the Kresling’s stretching at the faces, and we expect its behavior to closely follow kinematic models developed for paper Kresling structures rather than the stretchable and hyperelastic performance of softer materials such as silicone rubbers.

Recent work (Wu et al., 2021; Kaufmann et al., 2022) has demonstrated Kresling scales from a single millimeter to over 10 cm. Fabrication with a fused deposition modeling 3D printer imposes a practical limit on the scale. The structure’s sidewalls must be sufficiently thin to permit repeated contraction, but thick enough that they are reliably printed without leaks. Through experimentation, we determined that a wall thickness of 1 mm was the thinnest wall that delivered a yield above 75% on our equipment without pinholes or porous walls. We chose a scale with a visually obvious stroke between fully contracted and extended states of 2 cm or higher. Finally, the number of structure edges (*N*) may be four or more. However, a trade-off is present between increasing *N* and the stiffness of the structure, because more edges require more energy to deform the structure folds.

Taking these factors into account, we fabricated Kresling robots with *N* = 6, *R* = 3 cm, *L*_*e*_ = 4.1 cm, wall thickness *t* = 1 mm, and *λ* = 0.75, which correspond to an *α*_*e*_ of 30°. We set the *λ* value at the midpoint between fully-contracted (*λ* = 0.5) and fully-extended (*λ* = 1) values. These parameters generate a compliant structure with a maximum extension-contraction stroke of 31 mm.

**FIGURE 4:**
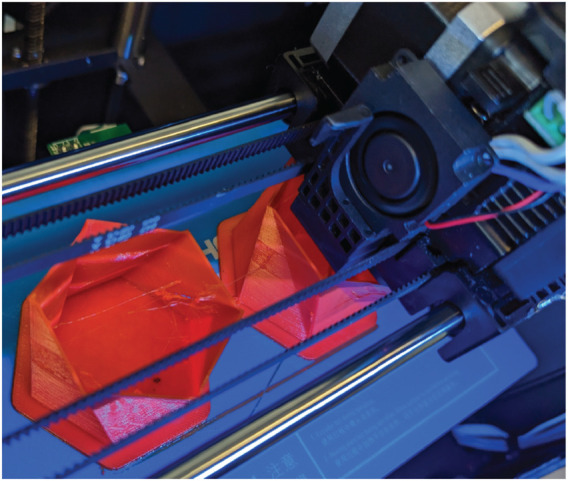
A photograph of the 3D printing process and two Kresling units. The print has progressed halfway.

To fabricate the Kresling robots, we programmatically generated a CAD (Fusion 360, Autodesk) model of the Kresling structure using a custom script that draws the bottom and sidewall faces given the design variables of *L*, *α*_*e*_, *N*, and *R* and sliced the model for 3D printing with FlashPrint slicer (v.5, FlashForge). We 3D printed (Creator Pro 2, Flash Forge) the Kresling structure with TPU filament (NinjaFlex Cheetah 95A) (Figure 4). Print settings were a 0.4 mm nozzle at an extruder temperature of 238 °C, platform temperature of 40 °C, layer height of 0.18 mm, and print speed of 30 mms^−1^.

To form the electrodes, we assembled a stack of copper and nickel plated polyester fabric (woven conductive fabric, 1168, Adafruit) and two-sided adhesive (double-sided adhesive sheets, Michaels, Inc.) with the backing remaining on the back side. We laid this stack onto a cutting mat (Standard Grip mat, Cricut, Inc.) with the back side facing the mat, then cut the electrode stack into the desired shape with an electronic cutting machine and knife blade (Maker 3, Cricut and Cricut Design Space software). We removed the backing from the electrode stack and manually adhered these electrodes to opposite faces of a valley fold using a thin, narrow line extruded on each sidewall during the print step to visually align electrode position. Three capacitors were symmetrically spaced radially around the *z*-axis. Each robot takes approximately three hours to print, adhere the electrodes, and seal.

To actuate and test the robot, we capped the structure with a TPU lid and cyanoacrylate glue (Loctite 495) and inserted pneumatic tubing through holes in the cap. Rigid plastic bases were bonded to the top and bottom faces of the Kresling actuator to enforce rigid bodies for kinematics and optical tracking. These bases also held the measurement cables at known locations relative to the robot. Figure 1A is a photograph of the robot mounted to a motion capture benchmarking stand.

The two-unit Kresling robot was fabricated with the same approach as the single-unit Kresling through the sealing step. Two individual Kresling units were stacked vertically with individual fluidic inputs for top and bottom units. The junction between the bottom and top unit has two holes for the fluidic inputs to enter the top unit. Two sets of test lead clips were added around this junction to hold half of the test leads for the bottom unit and half of the test leads for the top unit. One set of electrodes was adhered to the top and bottom unit each. These electrodes were measured through two channels on the capacitance to digital board to estimate the contraction of each unit.

### 4.2 Measurement and control setup

Actuator position was driven by a custom fluidic control board and a set of custom ROS Noetic (Quigley et al., 2009) nodes that published capacitance, calculated length, and set pulse width modulation (PWM) values. Data acquisition hardware (LabJack Pro T7) sourced a PWM signal to solenoids (Orange Coast Pneumatics) and the solenoids drove the actuation pressure within the Kresling from 101 kPA (full extension) to 80 kPa (full compression) absolute. Communication between the LabJack and ROS was enabled through the LabJack library for Linux (2019).

Capacitance was read at a rate of 90 Hz with a multi-channel capacitance-to-digital converter (FDC2214 EVM, Texas Instruments), which measures the resonance frequency of a circuit with a known value inductor, a known value capacitor, and the sense capacitor in parallel to calculate the capacitance value. In the single Kresling unit, capacitance was measured from three sets of electrodes adhered to alternating sidewalls and averaged across all three sets for each reading. Although the Kresling robot has six sets of sidewalls, we were limited to measurement on three sets for the single-unit robot and two sets for the two-unit robot due to limited channels on the measurement electronics.

To estimate the relationship between *L* and capacitance, the inverse kinematic model (Eq. 5) was combined with the FEA model of capacitance (*C*_*FEA*_) vs. *ξ* (Figure 3). This relationship was represented as a third-order polynomial function in the proprioception and control ROS nodes. Because humidity, local electromagnetic fields, and small variations in electrode placement affect capacitance and the match to the FEA model, we mapped *C*_*FEA*_ to the measured capacitance as *C*_*meas*_ = *κ* ^−1^ *C*_*FEA*_, where *κ* is a factor that scales *C*_*meas*_.

We selected a discrete-time implementation of proportional-derivative (PD) control due to its high stability and performance in this system. The value of the PWM signal at time *t* that was fed to the solenoid valves on the fluidic control board was

$${\text{PWM}}_{t}={\text{PWM}}_{t-1}+K_{p}\Delta L+K_{d}\frac{\Delta L}{\Delta t}$$ (6)

where PWM_*t*−1_ is the PWM value of the previous time step, *K*_*p*_ is the proportional gain constant, *K*_*d*_ is the derivative gain constant, Δ*L* is the error between the setpoint and the measured contraction, and Δ*t* is the time step. The proportional and derivative gain values were tuned by hand with values of 0.005 and 0.001, respectively. Before the setpoint was stepped, the single-unit Kresling was set to full extension and the initial capacitance was averaged over 5 s. This value is subtracted from subsequent capacitance values during the test to de-embed parasitic capacitance due to test cables.

The center of mass position and rotation of the actuator were measured using a commercial motion capture system (V120:Trio OptiTrack, and a custom Optitrack booth and Motive 2.3.7 and Motive 3.0, respectively) that served as the ground truth. Transform coordinate frames (TFs) were sent from Motive to ROS for additional processing through a Virtual Reality Peripheral Network (VPRN) interface (Bovbel 2017).

## 5 Results

### 5.1 Open-loop characterization

**FIGURE 5:**
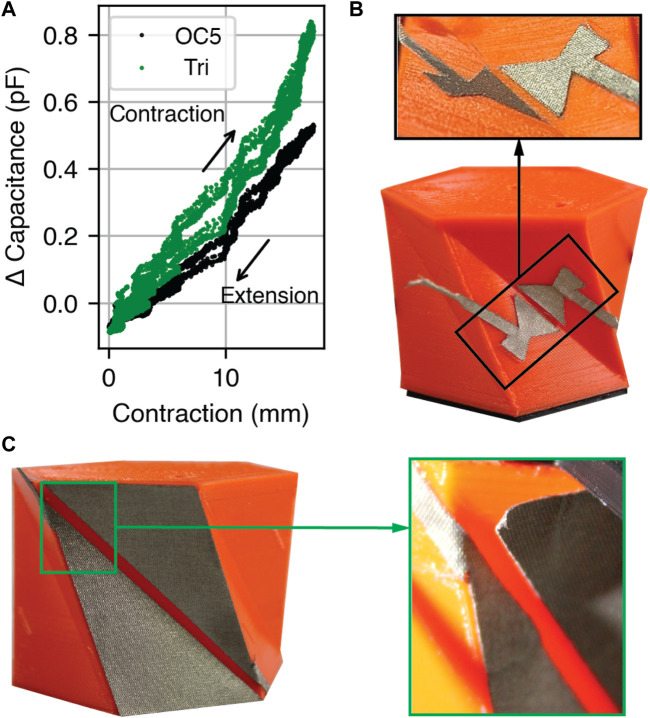
Contraction over 12 cycles to study cyclic loading performance. **(A)** Capacitance change of the triangular (“Tri.”) and optimized OC5 electrodes. **(B,C)** Photographs of the **(B)** OC5 and **(C)** triangular electrodes before actuation. *Insets:* electrodes after actuation. The free end of the OC5 electrode is visible and does not represent delamination.

We fabricated and compared the sensitivity of electrode OC5 and the triangular electrode by placing one set of each electrode type on the sidewalls of the same Kresling. We performed 12 cycles between 0 mm and 17 mm of contraction (Figure 5A). Because we cannot directly track fold angle through the motion capture system, we present the following results in terms of robot contraction along the *z*-axis.

The capacitance change per area between 0 mm and 17 mm of contraction is 0.22 pFcm^−2^ and 0.61 pFcm^−2^ for the triangular and OC5 electrodes, respectively. While the absolute sensitivity of the triangular electrode is higher, the OC5 electrodes yield 2.8 times higher capacitance per unit area.

After cyclic testing, we examined the electrodes for physical degradation. The OC5 electrodes, which sit in the center of the sidewall, showed no delamination from the robot body (Figure 5B) when compared to the pre-test condition. In contrast, the triangular electrodes wrinkled and delaminated at the corners (Figure 5C). This delamination was deemed unacceptable because loose electrodes may contact during contraction and yield inaccurate measurements. Therefore, the following tests are presented only with the OC5 electrodes.

**FIGURE 6:**
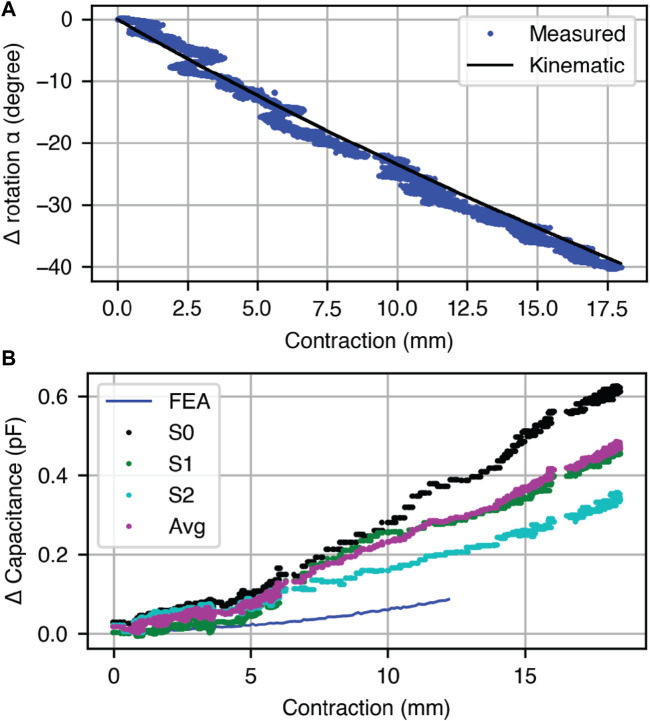
Open-loop response with contraction to verify the kinematic model of a single Kresling actuator. **(A)** Comparison of the observed Kresling contraction to the top face rotation *α* using the motion capture setup. **(B)** For three capacitive electrode pairs on a single Kresling, the measured capacitance change from full extension to full contraction. The average of the three sensor pairs is plotted and compared to the expected performance of the FEA model.

We investigated the relationship between *L* and *α* for a fabricated Kresling under open loop actuation to determine if the kinematic model that links *L* and *α* (i.e., Eq. 1) remains valid for Kresling units fabricated in TPU. Figure 6A is a plot of the kinematic model and measured data for a Kresling unit under open loop actuation from 0 to 17 mm contraction over 90 s. As the contraction increases, the rotation of the top face increases clockwise as predicted by the kinematic model. The root mean square error (RMSE) between measured *α* and kinematic model *α* is 1.5°.

During this open loop characterization, we also measured the relationship between contraction and capacitance for a Kresling robot with three sets of OC5 electrodes (S0, S1, and S2) (Figure 6B). These values are plot alongside the FEA model prediction for change in capacitance with contraction for OC5, *C*_*FEA*_. As contraction increases, the capacitance between all three sets of electrodes increases, and a difference in sensitivity is observed between the three sets of electrodes. The scales of the measured and modeled capacitance did not align, so we averaged the reading across three electrode sets at each contraction and fit a scale factor *κ* to this average, with a value of 0.26.

Finally, we measured the capacitance of an electrode set when the Kresling unit was at rest to determine the position error due to noise. Across 14,500 samples (120 s of data), the standard deviation was 0.01 pF, which corresponds to a length error of 1 mm at full extension, or 6% of maximum controlled contraction length.

### 5.2 Single-unit feedback control and performance under load

**FIGURE 7:**
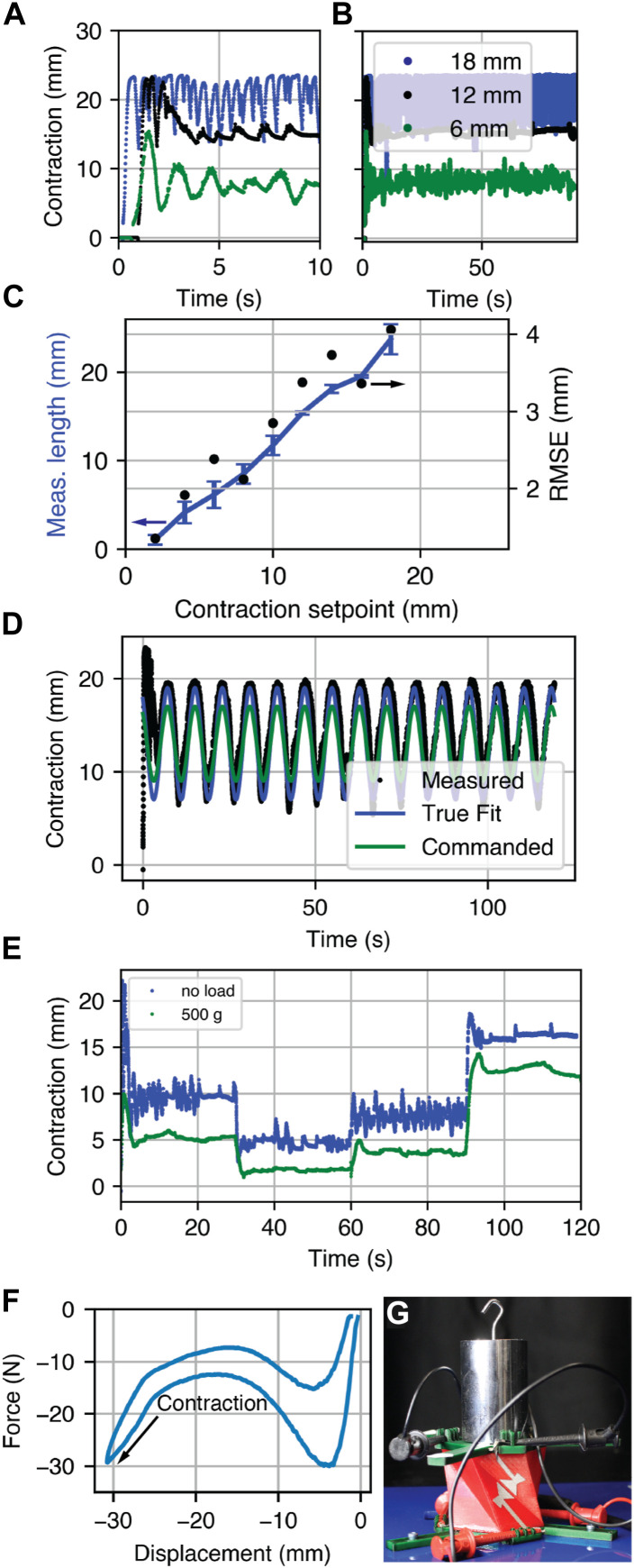
Feedback control response. **(A)** Three setpoints over 10 s and **(B)** over 90 s. **(C)** The actuator length across three trials and RMSE. **(D)** The response to a commanded sinusoidal input with the true fit representing a sinusoidal fit to the measured data and **(E)** control input steps for weighted and unweighted conditions at 8 mm, 4 mm, 6 mm, and 12 mm. **(F)** The force-displacement curve for a single Kresling unit. **(G)** A photograph of a single Kresling robot under a 500 g load during a position tracking test.

The setpoint was stepped from full extension to a contracted value under feedback control according to Eq. 6. The response of the Kresling robot to multiple commanded setpoints is shown in Figure 7, including at setpoints of 18 mm, 12 mm, and 6 mm (Figures 7A–C), time-varying sinusoidal tracking (Figure 7D), and multiple control setpoints stepped every 30 s (Figure 7E). RMSE and length are calculated from 20 s to 90 s during the test, and the error bars in Figure 7C represent standard deviation in length over three tests.

To determine the performance under load, we measured a force-displacement curve (Figure 7F) to the maximum compressed length of a single-unit Kresling completed with a force testing machine (Mecmesin iTest 2.5 kN through Emperor Force software), and placed a 500 g mass on the single-unit Kresling before the setpoint was stepped from 0 mm to a series of setpoints (Figures 7E, G). The RMSE is 3 mm and the Kresling contracts less than the experiment without the 500 g mass.

### 5.3 Two-unit feedback control

To demonstrate two-unit and two DOF Kresling robot control, we constructed a vertical stack of two Kresling units. This experiment is designed to demonstrate independent control of a multi-DOF Kresling robot, as an exemplar for how larger scale actuation is achievable by chaining together multiple units while still maintaining control of each. The fluidic input for each unit was independent and controlled by using a set of capacitive sensors on the top or bottom unit to estimate length and rotation of each unit and determine PWM input. Three target contractions and top face angles were set: *L*_*2DOF*_ = 68 mm, such that the contracted length is 14 mm and *α*_*2DOF*_ = 0°, and *L*_*2DOF*_ = 75 (7 mm contraction) with *α*_*2DOF*_ = −16.5° and *α*_*2DOF*_ = +16.5°.

**FIGURE 8:**
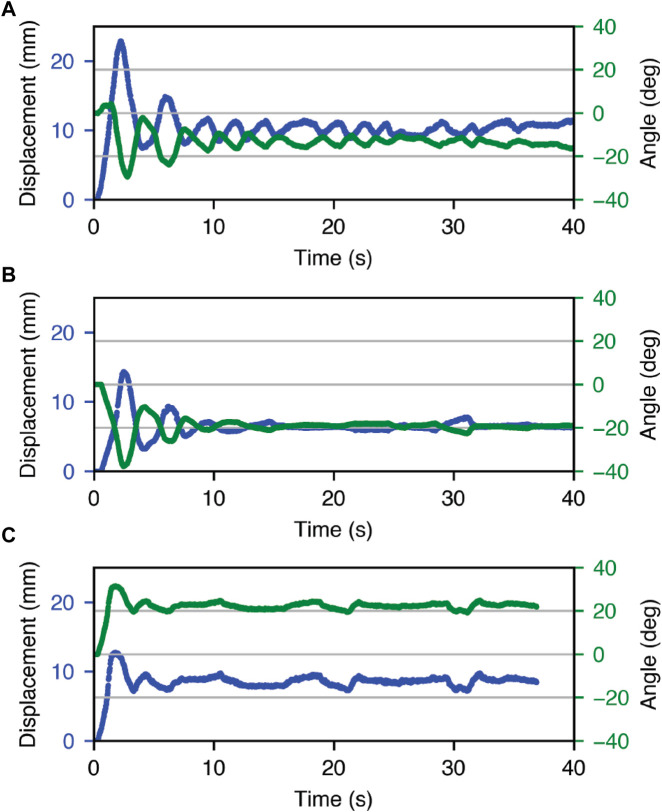
Feedback control response of the double Kresling robot, with target setpoints for contraction and *α* of **(A)** 14 mm and 0 0° **(B)** 7 mm and −16° **(C)** 7 mm and +16°.

Figure 8 is a plot of top face rotation and contraction for each commanded pose. In the first target conditions, the position overshoots before settling to its final value with contraction and angle RMSE of 3.6 mm and 13.6°, respectively, 30 s after the start of the test. In the second and third target conditions, the robot has contraction RMSE of 0.5 mm and 1.7 mm, respectively, with angle RMSE of 3.5° and 6.1°.

## 6 Discussion

The Kresling structure offers a rich testbed for investigating proprioceptive sensor performance in a soft robot. Integration of capacitive sensors onto the Kresling body permitted position control for contraction setpoints up to 17 mm, or 41% of the total robot length, with RMSE below 3 mm for setpoints less than 10 mm and below 4 mm for all setpoints. One source of error was the mapping from capacitance to length. The linear relationship observed in Figure 7C between length and setpoint has a slope higher than one, or a higher estimate of contraction. Increasing scale factor *κ* may improve position estimation.

We demonstrated that the triangle electrode had higher sensitivity than the optimized OC5 electrode but was not robust to repeated actuation. We attribute this performance to the mismatch between electrode strain and Kresling strain, particularly at the corners. The actuator visibly twists and stretches, causing the inextensible electrodes to delaminate. The OC5 electrodes are placed on regions of the sidewalls that remain relatively planar and unstretched, with only small concavity when a vacuum is introduced. This smaller deformation reduces the likelihood of delamination.

The sensitivities of each optimized electrode OC1-OC5 were on the same order of magnitude. We hypothesize that OC 5 was the optimal shape from the five electrodes due to lower capacitance at full contraction rather than higher capacitance at full extension. The small performance increase over the hexagon (OC1) and the inverted triangle (OC4) suggest that any electrode with sufficiently large sensitivity over the noise floor and located far from the triangular face corners may be suitable for closed-loop control.

Increasing setpoint beyond 17 mm of contraction caused instability, which is visible in Figure 7A as rapid oscillation around the setpoint. At this distance, the known bistable-like properties of the Kresling structure cause the robot to rapidly undergo a change in state for which the controller cannot compensate. Overshooting the setpoint causes the electrodes to make momentary contact, which results in maximum capacitance readings and therefore erroneously calculated short lengths. The system overcompensates as it perceives a much larger error than is present. This behavior is present in the first few seconds of the sinusoid following in Figure 7E. It follows a cyclic pattern; however, at larger contraction amplitudes, the system must overcome both the elastic restoring force present when the Kresling contracts to reach the set point and generates overshoot. To avoid this error in practice, we may simply restrict the contraction range of the contraction below 17 mm, thus preventing rapid change, overshoot, and contact.

The single-unit Kresling robot is able to withstand forces up to 30 N before full contraction, and the force-displacement curve demonstrates similar non-linear stiffness to behavior observed in Kresling robots fabricated from polymer films (Bhovad et al., 2019). Of note, due to strain energy stored in the TPU during contraction, there is no bistable point. This is a critical difference in this Kresling fabricated from an elastomer and previous observations of Kreslings fabricated in paper or polymer films.

The robot does demonstrate larger position error under load than without, and it undershoots the position target (i.e., less contraction than without the mass). Interestingly, this behavior opposes the expectation for an additional compressive force acting on the mass, i.e., the contraction should increase rather than decrease. We hypothesize that the response and decreased contraction is due to the large nearby metal, which will distort the relationship between capacitance and contraction. This response motivates further investigation of passive and active shielding approaches that screen the influence of the surrounding environment from the capacitive sensors.

We established the ability to independently control robot length and top face rotation using sets of capacitive electrodes on the top and bottom Kresling units. The contraction setpoint errors were in line with the results observed from the single-unit Kresling error (3.6 mm vs. 3.4 mm, respectively at 14 mm contraction), and the angle errors for one actuated unit within the two-unit robot were slightly larger (3.5° and 6.1° vs. 2.1°) than for the single-unit Kresling. The angle error for the two-unit Kresling when both the top and bottom units were actuated was appreciably larger (13.6°). During actuation, the bottom Kresling appears to tilt and twist when the top Kresling unit is actuated. This behavior may be attributed to a non-ideality in the bottom unit sidewall thickness that creates bending under actuation.

The nonlinear capacitance-length relationship, non-rigid deformation, and bi-stable behavior add complexity to feedback control and provide additional motivation for feedback control over open-loop control. While open-loop control is more common in soft robotics, it also requires a much finer degree of system identification for accurate control. Therefore, we view proprioceptive feedback as a crucial capability for emerging soft robotics.

## 7 Conclusion

Applying proprioceptive position sensing to control actuator position is an ongoing challenge in soft robotics. To address this challenging problem, we demonstrated a Kresling origami-inspired, 3D printed, fluidically actuated robot with capacitive sensors integrated onto the sidewall faces. Through optimizing the geometry of the capacitive electrodes, we maximized the sensitivity at large fold angles. The observed kinematics of the Kresling rotation and contraction show strong agreement with a linear model of changing capacitance along the sidewall folds. We demonstrated the utility of our sensing and modeling to achieve varying conformal states of the Kresling along its range of motion.

We further demonstrated the ability to transition between setpoint states and achieved less than 4 mm setpoint error across a setpoint to 17 mm. This minimized error allowed us to achieve complex motion patterns such as tracking a sine wave. We also observed the Kresling performance to achieve desired contraction under external loading. The success of the 1DOF control was expanded to a 2DOF system of stacked Kreslings, where we achieved independent control of contraction and rotation angle.

This work constitutes a significant step towards reliable, low-cost proprioceptive sensing for origami robots. Building on the 2DOF robot, we intend to explore the pantheon of achievable robot morphologies by using the Kresling as the base unit. This will include actuators with controllable bending. Joining several sensorized Kresling structures will enable complex motion strategies that include crawling and movement in 6-DOF pose space. Other areas of future work include improving the electrode design and manufacturing, fabrication approaches to reduce capacitance variations between electrode pairs, and investigating different control approaches. Future research in multi-Kresling robots will also explore passive and active shielding approaches in future work to reduce environmental interference in the electrodes.

The results of this work demonstrate that capacitive sensing is a promising and adaptable technique for proprioceptive state estimation in soft robotics.

## Data Availability

The original contributions presented in the study are publicly available. This data can be found here: http://hdl.handle.net/2047/D20603056 and https://parses-lab.github.io/kresling_control/.
